# Inflammation down-regulates CYP3A4-catalysed drug metabolism in hemodialysis patients

**DOI:** 10.1186/s40360-018-0221-6

**Published:** 2018-06-25

**Authors:** Hadi Molanaei, Abdul Rashid Qureshi, Olof Heimbürger, Bengt Lindholm, Ulf Diczfalusy, Björn Anderstam, Leif Bertilsson, Peter Stenvinkel

**Affiliations:** 1 Division of Renal Medicine and Baxter Novum, Department of Clinical Science, Intervention and Technology, Karolinska Institutet, Karolinska University Hospital, SE-141 86 Stockholm, Sweden; 2 Division of Clinical Chemistry Department of Laboratory Medicine, Karolinska Institutet, Karolinska University Hospital, Stockholm, Sweden; 3 Division of Clinical Pharmacology, Department of Laboratory Medicine, |Karolinska Institutet, Karolinska University Hospital, Stockholm, Sweden

**Keywords:** Inflammation, CYP3A4, hemodialysis, Drug metabolism, quinine

## Abstract

**Background:**

Recent studies indicate that inflammation may also affect CYP3A4 activity. Associations of CYP3A4-mediated metabolism of quinine, with inflammatory biomarkers were investigated in patients undergoing maintenance hemodialysis (HD).

**Methods:**

A single dose of 100 mg quinine was given to 44 HD patients and the plasma concentration of quinine and its metabolite 3-OH-quinine were measured 12 h after drug intake. The ratios of quinine/3-OH-quinine and 4β-OH-cholesterol/cholesterol were used as markers of CYP3A4 activity. Inflammatory biomarkers, high-sensitive CRP (hsCRP), pentraxin 3 (PTX3) and orosomucoid were followed during 4 weeks prior to quinine administration.

**Results:**

The quinine/3-OH-quinine ratio correlated with median concentrations of hsCRP (Rho = 0.48; *p* = 0.001) and orosomucoid (Rho = 0.44; *p* = 0.003), and also with interleukin-6 at 12 h after drug intake (Rho = 0.43; *P* = 0.004) but not PTX3. In multivariate regression analysis, the correlation between CYP3A4 activity and median hsCRP remained borderline significant (*p* = 0.05). 4β-OH-cholesterol/cholesterol ratio correlated with quinine/3-OH-quinine (*p* = 0.008), but not with any of the inflammation markers.

**Conclusions:**

The association between CYP3A4 activity and inflammatory biomarkers suggest that the activity of CYP3A4 is reduced by inflammation in HD patients. Further studies are needed to confirm this finding and to assess to what extent magnitude and duration of inflammation as well as the microbiota affect drug metabolism.

## Background

Chronic kidney disease (CKD) patients are at a high risk for drug side effects due to accumulation of drugs, which normally are excreted via the kidneys. It is believed that drugs that are metabolized by the liver are safe to prescribe in normal doses to end-stage renal disease (ESRD) patients. However, emerging data show that kidney failure itself can affect enzymes and drug transporters [1, 2]. Thus, the concentration of many drugs that normally are metabolized by the liver increases significantly in CKD patients. It is also possible that renal failure modifies drug interactions, including the affinity of some drugs to their receptor, the number of receptors, and the cell responses upon receptor activation. Taken together, all these factors contribute to a high risk of both under- and overdosing of drugs in this patient group.

A common feature of the uremic phenotype that may affect drug metabolism is persistent inflammation [3, 4]. Inflammation has been shown to reduce the activity of both drug metabolizing enzymes and transporters [5–8]. Pro-inflammatory cytokines are thought to be principal mediators of the impact of inflammation on these enzymes and transporters [6]. The mechanism of this inhibitory effect is not clear so far but the transcriptional suppression is thought to be a major factor in the downregulation of many cytochrome P450s and there could be multiple posttranscriptional effects involved as well [6]. Presence of the above mentioned factors increases the risk of side effect and drug interactions even more, especially in ESRD patients who on a daily basis take > 20 pills per day [9]. No doubt, such a large number of prescribed pills contributes to unintentional poor compliance in this patient group and adherence to drug prescriptions is in general much lower in dialysis patients than anticipated [10, 11].

By using alprazolam as a test drug we previously showed that a low degree of inflammation significantly reduced the activity of CYP3A4 [12], which is the most important hepatic drug-metabolizing enzyme accounting for the metabolism of almost 50% of all drugs currently used [13, 14]. Thus, any changes in its activity may alter drug metabolism of a number of drugs, leading potentially to serious consequences in terms of effects of drugs, risk of side effects and, ultimately, quality of life, safety and survival of the dialysis patient. Although the exact mechanism of enzyme inhibition by inflammation is not clear, previous studies have discussed the possible causative effect of inflammatory markers, such as C-reactive protein (CRP) on the activity of CYP3A4 [15, 16]. Moreover, the inflammatory marker orosomucoid has been shown to bind drugs and could thereby potentially raise their concentration in plasma [17]. Thus, this effect could be misinterpreted as a decreased activity of the drug-metabolizing enzyme.

The aim of this study was to investigate the effect of inflammation on the activity of CYP3A4. We investigated 44 prevalent hemodialysis (HD) patients using quinine (substrate of CYP3A4) as a test drug to investigate the effect of inflammation over four weeks on drug metabolism. Quinine is an anti-malaria drug with spasmolytic effects that commonly is prescribed in low doses (100–250 mg) to HD patients suffering leg cramps.

## Methods

### Patients

Fifty-four prevalent HD patients with no or minimal residual renal function agreed to participate in the study. Ten patients did not complete the study, see below. Baseline characteristics of the patients are shown in Table 1. All patients underwent three HD sessions per week (3.5–5.0 h/session) using high-flux polysulphone membrane dialyzers. All the patients were included in the study during between Nov. 2011 and March 2012. They were followed up during five weeks from the initiation of the study and until the study was completed.

**Table 1:** Demography and laboratory parameters

Age, years	71 (61–77)
Female/male, n	11/33
Dialysis vintage, months	36 (20–72)
Body mass index, kg/m^2^	25.0 (23.0–29.5)
Diabetes mellitus, n	23
Cardiovascular disease, n	30
Primary kidney disease, n	
*Diabetes mellitus*	12
*Chronic glomerulonephritis*	5
*Nephrosclerosis*	7
*Polycystic kidney disease*	4
*Vasculitis*	2
*Other or unknown disease*	14
Medications, n	
*Beta-blockers*	25
*ACEi/ARBs*	9
*Statins*	15

Data presented as number of patients (n), or as median and interquartile range (IQR). *Abbreviations*: *ACEi* angiotensin converting enzyme inhibitor, *ARBs* angiotensin receptor blockers

### Study design

Study design and time points for blood samples taken during the study are shown in Table 2. Four patients decided not to continue with the study. One patient was excluded due to a prolonged infectious episode. Two other patients were excluded from the study due to undetectable levels of both quinine and 3-OH-quinine in their plasma indicating that they did not take the test drug. Two patients died before the end of the study and another patient underwent kidney transplantation before completing the study. Each patient was given 100 mg quinine (Kinin®100 mg) orally in the evening before the day of a regular dialysis day. Samples of 10 ml peripheral blood were drawn into EDTA containing tubes at the beginning of the dialysis session. Plasma was separated and stored frozen at − 80 °C until analysis. The concentration of quinine and its metabolite 3-OH-quinine was measured in the blood sample collected 12 h after drug intake at the beginning of the dialysis. The ratio of quinine and 3-OH-quinine was used as a marker for CYP3A4 activity [18]. The levels of hsCRP, orosomucoid, and pentraxin 3 (PTX3) were measured weekly before dialysis sessions during the four weeks prior to the drug test (Table 2). Measurements were repeated in plasma samples collected 12 h after drug intake concomitantly with measurements of 4β-OH-cholesterol, cholesterol, hemoglobin, albumin, iron, N-terminal-pro-Natriuretic peptide (NT-pro-BNP), parathyroid hormone (PTH) and IL-6 (Table 2). No side effects of the drug were reported. None of the patients was treated with CYP3A4 inhibitors or inducers. Exclusion criteria were signs of local or systemic infection such as chill, fever, fatigue, increasing white blood counts or median hsCRP> 50 mg/L.

**Table 2:** Study design, patient flow and samples taken during the study

Week 1 *n* = 54^a^	Week 2 *n* = 50^b^	Week 3 *n* = 49^c^	Week 4 *n* = 47^d^	Week 5 (n:44)^e^	
				Day 1	Day 2
Inclusion hsCRP, PTX3, orosumucoid	hsCRP, PTX3, orosomucoid	hsCRP, PTX3, orosomucoid	hdCRP, PTX3, orosomucoid	Administration of 100 mg quinine	hdCRP, PTX3, IL-6 and orosomucoid Hb, P-Iron, P-albumin, NT-pro-BNB and PTH 4β-OH-Cholesterol and P-Cholesterol Concentrarion of quinine and 3-OH-quinine

^a^At screening, ^b^Four patients decided to withdraw their consent and did not continue with the study, ^c^One patient underwent kidney transplantation, ^d^Two patients died before completing the study, ^e^Three patients were excluded; one due to an infectious episode and two because of undetectable levels of quinine and 3-OH-quinine in plasma

### Analyses

The concentrations of quinine and its metabolite 3-OH-quinine were determined by high-performance liquid chromatography with tandem mass spectrometric detection (UPLC-MS/MS) following sample separation by protein precipitation with acetonitrile containing internal standards [18]. A 100 μL volume of the sample was protein precipitated with 200 μL of the internal standard solution. The extract was injected into the UPLC-MS/MS system. Separation of the analytes was achieved on an Acquity UPLC BEH C18-column (2.1 × 50 mm 1.7 μm), using gradient run with mobile phase A (11 mM ammonium formate) and mobile phase B (0.1% formic acid in acetonitrile). The analytes were detected using a Micromass Quattro Primer XE mass spectrometer operating in positive electrospray ionization (ESI) mode utilizing selected reaction monitoring (SRM) for the transitions 325 → 160 m/z for quinine and 341 → 160 m/z for 3-OH-quinine. IL-6 was analyzed in serum by an immunometric assay on an Immulite 1000 Analyzer (Siemens Healthcare, Los Angeles, CA, USA) according to the instructions of the manufacturers. PTX3 was analyzed in EDTA plasma with sandwich ELISA from R&D systems (Abingdon, UK). Cholesterol was determined on a Roche/Hitachi Modular instrument using a commercial enzymatic method (Cholesterol CHOD-PAPP, Roche Diagnostics, GmbH, Mannheim, Germany). The between-day variation was 1.3% at 5 mmol/L. Orosomucoid, hemoglobin, iron, NT-pro-BNP, albumin, and hsCRP in plasma were measured by validated routine methods used by the accredited clinical chemistry laboratory at Karolinska University Hospital, Stockholm. Plasma 4β-OH-cholesterol was determined by isotope-dilution gas chromatography-mass spectrometry, using deuterium labeled 4β-OH-cholesterol as internal standard as described previously [19, 20]. The within-day variation was 4.5% and the between-day variation was 8.2%. The lower limit of quantification was 4.8 ng/mL and the method was linear up to 600 ng/mL. The ratio of 4β-OH-cholesterol/cholesterol was used as an additional marker of CYP3A4 activity [20].

### Statistical methods

All variables are expressed as mean ± SD or as median (25th and 75th percentile), unless otherwise indicated. Statistical significance was set at the level of *p* ≤ 0.05. Non-parametric Spearman’s rank correlation analysis was used to determine associations between various variables. We used one way non-parametric ANOVA to study variation of CRP, PTX3 and orosomucoid. Determinants of ratio quinine/3-0H-quinine were explored using multivariate linear regression analysis. All statistical analyses were performed using statistical software SAS version 9.4 (SAS Campus Drive, Cary, NC, USA).

## Results

### Characteristics of the patients

Laboratory data in the 44 HD patients on the day of investigation, i.e., 12 h after intake of a single dose of 100 mg quinine (“Day 2” in Table 2), and median values for hs-CRP, orosomucoid and PTX3, calculated from five samples taken during 4 weeks prior to the study plus on Day 2, are shown in Table 3.

**Table 3:** Laboratory values in 44 HD patients on the final day of investigation (Week 5, Day 2; see Table 2); however, values for inflammatory markers, hsCRP, orosomucoid and PTX3, are shown as median values over 4 weeks plus the final day of investigation

Laboratory parameters	
hsCRP, mg/L ª	4.5 (1.5–14.0)
Interleukin-6, pg/ml	6.0 (3.0–10.6)
Orosomucoid, g/L ª	1.0 (0.8–1.3)
Pentraxin 3, ng/ml ª	1.3 (1.1–2.0)
NT-pro-brain natriuretic peptide, ng/L	6330 (1285–21,830)
Parathyroid hormone, ng/L	322 (151–587)
Albumin, g/L	33 (32–36)
Iron, μg/L	10 (8–13)
4β-OH-Cholesterol, ng/ml	26 (14–26)
Cholesterol, mmol/L	3.9 (3.4–4.7)
Urea reduction rate, %	74 (70–78)
Hemoglobin, g/L	115 (106–121)

Data presented as median and interquartile range (IQR). ª Median values for high sensitive (hs) CRP, orosomucoid and pentraxin-3 are calculated from the five samples taken during 4 weeks prior to the study plus the last day of the study. The rest of the parameters are sampled at day 2 of week 5

### Correlations

**Fig. 1:**
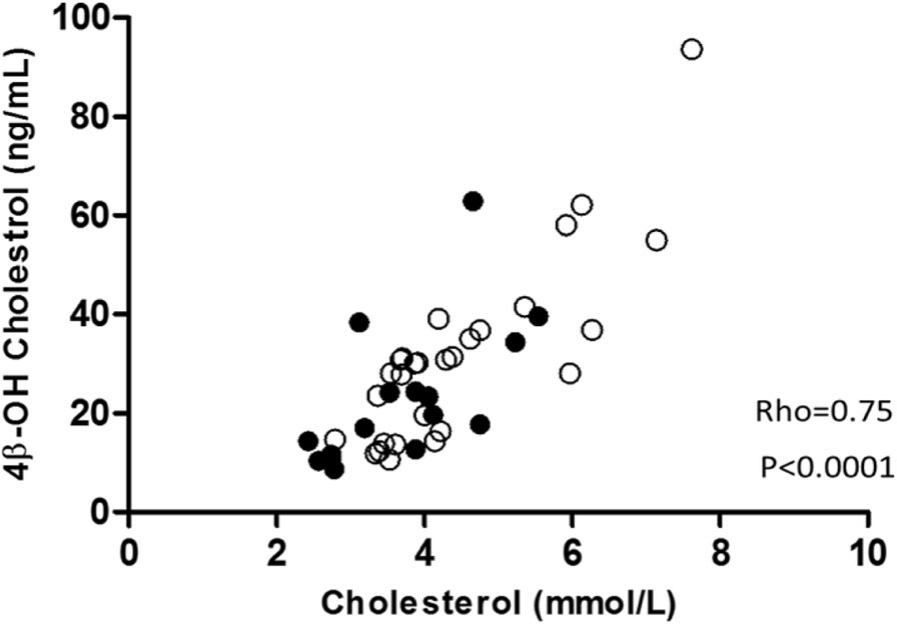
Correlation between 4-beta-OH-cholesterol and cholesterol. Closed circles represent 15 patients treated with statins and open circles represent 29 patients without statin treatment

**Fig. 2:**
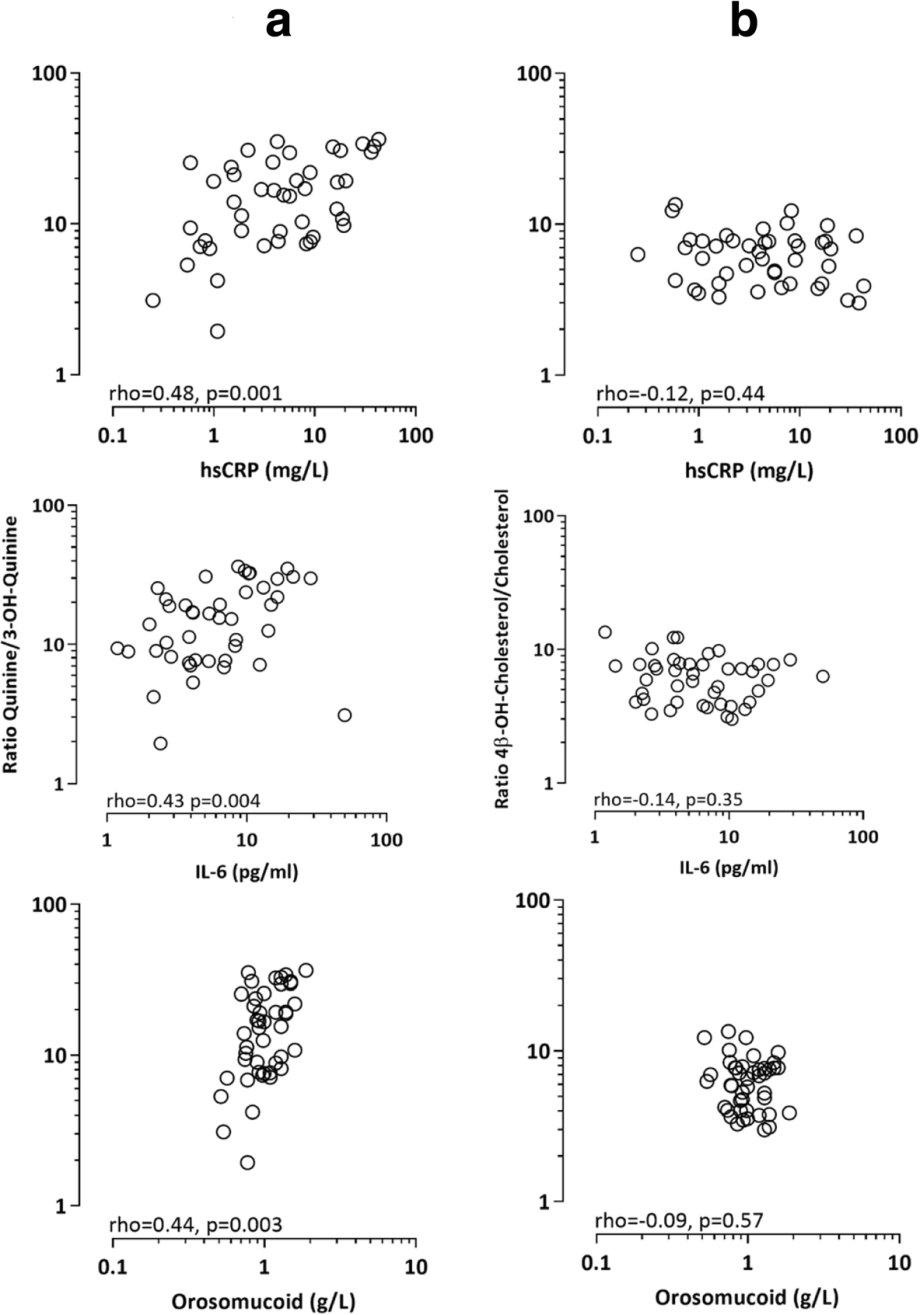
Correlation between CYP3A4 activity, expressed as the ratio of quinine/3-OH-quinine (**a**) and 4β-OH-cholesterol/cholesterol (**b**), respectively, and median concentrations of hsCRP and orosomucoid as well as with a single measure of IL-6 (on Day 2 at week 5 of the study)

Significant correlations were observed between plasma albumin and both preceding time on dialysis, dialysis vintage (Rho = 0.37; *p* = 0.013) and age (Rho = − 0.39; *p* = 0.0085). As expected, inflammatory markers correlated with each other: median orosomucoid vs. median hsCRP (Rho = 0.83; *p* < 0.001), median orosomucoid vs. IL-6 (Rho = 0.46; *p* = 0.0016) and IL-6 vs. median hsCRP (Rho = 0.45; *p* = 0.0023). A significant correlation was also observed between 4-beta-OH-cholesterol and cholesterol (Rho = 0.75; *p* < 0.0001) (Fig. 1). As 15 of the 44 patients were treated with statins, we investigated this correlation in patients with or without statins (Fig. 1). Significant correlations were observed both in patients on statins (Rho = 0.67; *p* = 0.006) and not on statins (Rho = 0.83; p < 0.0001) (Fig. 1). Significant correlations between CYP3A4 activity (expressed as ratio of quinine/3-OH-quinine) and median hsCRP (Rho = 0.48; *p* = 0.001), median orosomucoid (Rho = 0.44, *p* = 0.003) and IL-6 at day 2 at the end of week 5 (Rho = 0.42; *p* = 0.004) were observed (Fig. 2a). Median values of hsCRP, PTX3 and orosomucoid were calculated from the measurements four weeks prior to the study plus the values taken at the last day of the study. We observed no statistically significant variations (non-parametric ANOVA) of CRP, PTX3 and orosomucoid during the study period (data not shown). Also the last value (samples taken at the same day as quinine) of hsCRP (Rho = 0.59; *p* < 0.001) and orosomucoid (Rho = 0.45; *p* = 0.002) correlated to the ratio of quinine/3-OH-quinine. We did not observe any correlation of the ratio of quinine/3-OH-quinine with NT-pro-BNP (Rho = 0.15; *p* = 0.332,) and plasma albumin (Rho = − 0.18; *p* = 0.237), respectively. A significant correlation (Rho = − 0.40; *p* = 0.008) was also found between ratio of quinine/3-OH-quinine and 4β-OH-cholesterol/cholesterol; a marker of CYP3A4 activity. No correlations were observed between the ratio of 4β-OH-cholesterol/cholesterol and median hsCRP (Rho = − 0.12; *p* = 0.44), median orosomucoid (Rho = − 0.09; *p* = 0.57) and IL-6 (Rho = − 0.14; *p* = 0.35) (Fig. 2b), respectively. We did not find any correlation between median PTX3 and CYP3A4 activity expressed as either quinine/3-OH-quinine (Rho = − 0.17; *p* = 0.26) or 4β-OH-cholesterol/cholesterol (Rho = − 0.21; *p* = 0.16). Sixteen participants in the study where treated with statins which is considered as an inhibitor of CYP3A4 but our results showed no significant difference between the participants with or without statin (P: 0.96).

### Multivariate analysis

The association between CYP3A4 activity expressed as quinine/3-OH-quinine and hsCRP showed a trend to be significant (β = 0.44; *p* = 0.05) in a multivariate analysis after full adjustment for age, gender, diabetes mellitus, dialysis vintage, PTH, orosomucoid and medication with angiotensin-converting enzyme inhibitors (ACEIs), angiotensin receptor blockers (ARBs), beta-blockers or statins (Table 4).

**Table 4:** Multivariate linear regression models for determinants of the ratio quinine/3-0H-quinine in 44 prevalent hemodialysis patients. Data are expressed as adjusted r^2^, beta (β) and significance

	Unadjusted (β, *P*) (r^2^ = 0.23)	Model 1 (β, *P*) (r^2^ = 0.27)	Model 2 (β, *P*) (r^2^ = 0.27)	Model 3 (β, *P*) (r^2^ = 0.21)
hsCRP (mg/L)	0.49 (**0.001**)	0.52 (**0.001**)	0.54 (**0.001**)	0.44 (**0.05**)
Age (years)		0.02 (0.90)	−0.002 (0.98)	− 0.03 (0.85)
Gender (female)		0.28 (**0.04**)	0.27 (0.07)	0.30 (0.07)
Diabetes mellitus		−0.13 (0.32)	−0.18 (0.22)	− 0.14 (0.44)
Vintage (months)		−0.11 (0.42)	−0.10 (0.50)	− 0.08 (0.62)
Betablockers			0.14 (0.38)	0.12 (0.46)
ACEi/ARBs			−0.21 (0.19)	−0.21 (0.21)
Statins			0.18 (0.31)	0.18 (0.31)
PTH (ng/L)				−0.01 (0.93)
Orosomucoid (g/L)				0.14 (0.56)

*Abbreviations***:** *hsCRP* High sensitivity C reactive protein, *ACEi* Angiotensin converting enzyme inhibitors, *ARBs* Angiotensin receptor blockers, *PTH* Parathyroid hormone

Bold text indicate significance levels

## Discussion

In the present study, a single dose of 100 mg quinine was given to 44 ESRD patients undergoing maintenance HD and plasma quinine and its metabolite 3-OH-quinine were measured after 12 h immediately prior to the next HD session. Patients with signs of inflammation - according to weekly measurements of several biomarkers of inflammation over 4 weeks prior to the investigation – had a higher quinine/3-OH-quinine ratio, indicating decreased CYP3A4 activity, suggesting that the activity of CYP3A4 is reduced by inflammation in HD patients.

As kidney failure can alter the pharmacokinetics of many drugs at different levels [21, 22], drug dosing has always been a challenge in this vulnerable patient population [23]. The absorption of orally administered drugs in the gastrointestinal system may be affected by reduced gut motility, increased pH, increased paracellular transport across the intestinal epithelium [24] and reduced activity of drug metabolizing enzymes and transporters [1, 25]. This may result in increased rate of absorption and consequently increased bioavailability of drugs. The absorbed drugs need to be bound to albumin or other plasma proteins during the transport of drugs to the target organ or liver. However, as the concentrations of plasma proteins, including albumin, in general are decreased in ESRD patients, protein binding of drugs is reduced, and the free circulating concentration of the drug may therefore increase [26]. Furthermore, the uremic milieu per se could reduce the non-renal elimination of drugs by affecting the function of drug metabolizing enzymes and transporters leading to even larger risk of drug accumulation and drug intoxication [1].

As dialysis patients are often subjected to polypharmacy this increases the risk of drug-drug interactions [9, 27]. Furthermore, the concentration of circulating drugs is not only affected by the changes in drug metabolism and pharmacokinetics, but also by the dialysis treatment per se. The dialyzability of a drug depends on several factors, such as molecular weight, protein binding, volume of distribution, blood and dialysis flow rates during the dialysis treatment, and type of the dialysis membrane [22, 28]. Another important observation is that HD is reported to increases the metabolic activity of CYP3A4 suggesting that dialyzable uremic toxins may inhibit the activity of this enzyme [29, 30]. Thus, nephrologists need to consider a myriad of factors when a drug is prescribed to a dialysis patient. Further, as inflammation may alter the activity of drug metabolizing enzyme and transporters [7], this condition which is common in dialysis patients may add to the difficulties to prescribe drugs [28].

So far most studies on the impact of inflammation on drug metabolism have been conducted in animal models [7, 31]. In the current study, we studied pharmacokinetics of quinine, a substrate for CYP3A4, in a group of HD patients. Quinine is commonly used in dialysis wards against leg cramps in HD patients during the dialysis treatment and the risk of side effects is small for current doses prescribed to HD patients (100-250 mg). Our results show that CYP3A4 activity associate to biomarkers of inflammation. Since the already complicated drug metabolism in dialysis patients may be affected also by systemic inflammation, this adds novel challenges to correct drug dosing in this inflamed patient population [32]. Further, as Shah and Smith [33] reported that phenoconversion of drug metabolizing enzymes may be an important modifier of drug metabolism, the scenario may be even more complicated. Since inflammation may induce phenoconversion [34] this implies that genetically extensive metabolizers could be converted to a phenotypic poor metabolizer. Clearly, we need to individualize drug dosing to reduce the complications related to drug side effects and interactions. For this purpose we need to identify which factors in addition to traditionally known factors that affect pharmacokinetics and pharmacodynamics of drugs.

The results of the present study should be considered in light of the following strengths and caveats. The careful repeated monitoring of inflammation biomarkers during four weeks preceding the pharmacokinetic study of quinine strengthens the ascertainment of the inflammatory burden of the investigated patients. Some caveats deserve mentioning. As the sample size is rather small, the results need to be confirmed in larger cohorts. In a prior study, using alprazolam as a test drug, we demonstrated that inflammation associated to reduced activity of CYP3A4 in another cohort of HD patients [12]. Since orosomucoid can be considered as a confounder due to its ability to bind drugs [17], thereby raising the plasma concentration of quinine, this could lead to misinterpretation of reduced CYP3A4 activity. However, the correlation between quinine/3-OH-quinine and hsCRP showed a trend to being significant (*p* = 0.05) after multivariate regression analysis (Table 4) implying that inflammation per se affect the activity of CYP3A4. In the present study, no correlation between CYP3A4 activity and PTX3 was observed. However, it has been reported that PTX3 may primarily reflect endothelial dysfunction rather than systemic inflammation [35]. Another limitation is that the impact of accumulation of uremic toxins and its putative effects on drug functions and metabolism was not assessed.

Although we report a significant correlation between CYP3A4 activity expressed as quinine/3-OH-quinine and the ratio of 4β-OH-cholesterol/cholesterol; the latter being another marker for CYP3A4 activity [36], no correlation between the inflammatory biomarkers and the ratio of 4β-OH-cholesterol/cholesterol was observed. This finding is in accordance to our prior study using alprazolam as test drug [12]. Although 4β-hydroxycholesterol is considered to be a useful marker for CYP3A4 activity in healthy individuals, this marker may not be suitable for detecting CYP3A4 activity in HD patients. One reason could be that this cholesterol metabolite is very slowly eliminated after induction and therefore the relatively faster variation of the degree of inflammation may not be reflected by changes in metabolism of the slowly eliminated 4β-OH-cholesterol [12]. CYP3A accounts for 80% of total P450 content in intestines [37], and although the CYP3A content in intestine is only about 1% of the amount in the liver, its predominance in human intestine can lead to several fold more efficacy of the enzyme in intestine compared to liver [38, 39]. Indeed, the intestine is suggested to be of equal or even greater importance than liver for metabolism of drugs [40]. Furthermore, since the inflamed uremic milieu is associated with changes in gut microbiota [41], the discrepant findings with regard to the relation of quinine/3-OH-quinine and 4β-OH-cholesterol/cholesterol with inflammation could imply a role of uremic dysbiosis on the pharmacokinetic profile [42]. In a previous study in 440 healthy subjects representing three major populations in Africa, Asia and Europe, the coefficient of correlation between 4β-OH-cholesterol and cholesterol was low (*R* = 0.30), but significant (*p* < 0.0001); i.e. only 9% of the variation in 4β-OH-cholesterol concentration was due to the variation in cholesterol concentration [43]. Thus, a major determinant of the level of 4β-OH-cholesterol might be the CYP3A4 activity and not the concentration of the substrate i.e., cholesterol. In the present study, we found a much stronger correlation (rho = 0.75); fairly independent of statin treatment (Fig. 1). In our previous study using alprazolam as a marker of CYP3A4 activity, we also found a fairly high coefficient of correlation between 4β-OH-cholesterol and cholesterol (Rho = 0.58; *p* = 0.0018) (calculated from data of ref. [12]). In these two studies on HD patients the variation in 4β-OH-cholesterol concentration is determined to a pronounced extent by cholesterol (56% in the present study and 34% in [12]), which is higher than the 9% reported in healthy subjects [43]. We propose that 4β-OH-cholesterol/cholesterol is a better marker of CYP3A4 activity in healthy subjects while it is an inadequate marker in HD patients which could explain the absence of a relationship between CYP3A4 activity measured by 4β-OH-cholesterol and markers of inflammation in two independent groups of HD patients investigated by our group. Further Björkhem-Bergman et al. [44] have shown that whereas statin treatment had no effect on the hepatic CYP3A mRNA content, it significantly reduced 4β-OH-cholesterol, while there was no significant effect on the 4β-OH-cholesterol/cholesterol ratio [45]. An earlier in vitro study showed that the CYP3A4 enzyme is saturated at a cholesterol concentration of 100 μM [19]. Both 4β-OH-cholesterol and cholesterol are transported in lipoproteins in the circulation [19]. These data indicate that during statin treatment it is mainly the cholesterol-dependent lipoprotein binding capacity in the circulation that will determine the 4β-OH-cholesterol concentration in plasma, rather than a direct effect on the hepatic CYP3A4 enzyme. A disturbed cholesterol-dependent lipoprotein binding capacity in HD patients may also be operative [46]. Taken together, our results suggest that the ratio 4β-OH-cholesterol/cholesterol rather than 4β-OH-cholesterol alone, is the preferred measure of CYP3A4 activity.

## Conclusion

In summary, a higher degree of inflammation associates with decreased activity of CYP3A4 in HD patients. Further studies are needed to find out if this consequence of inflammation will have a clinical significant impact on risk of drug interactions and side effects in dialysis patients.

## Abbreviations

ACEi: Angiotensin converting enzyme inhibitor
ARBs: Angiotensin receptor blockers
CKD: Chronic kidney disease
CRP: C-reactive protein
CYP3A4: Cytochrome P450 3A4
ESRD: End stage renal disease
HD: Hemodialysis
hsCRP: High sensitive CRP
NT-pro-BNP: N-terminal pro-Brain Natriuretic peptide
PTX3: Pentraxin 3
